# Supplementary material for: Shaping the Physicochemical, Functional, Microbiological and Sensory Properties of Yoghurts Using Plant Additives
Source: Foods. 2023 Mar 17;12(6):1275. doi: 10.3390/foods12061275 (PMC10048245; doi:10.3390/foods12061275)
Supplement: Supplementary file 1 [file foods-12-01275-s001.zip › Figure S1.PRISMA_2020_flow_diagram_new_SRs_v1.docx.pdf]

PRISMA 2020 flow diagram for new systematic reviews which included searches of databases and registers only

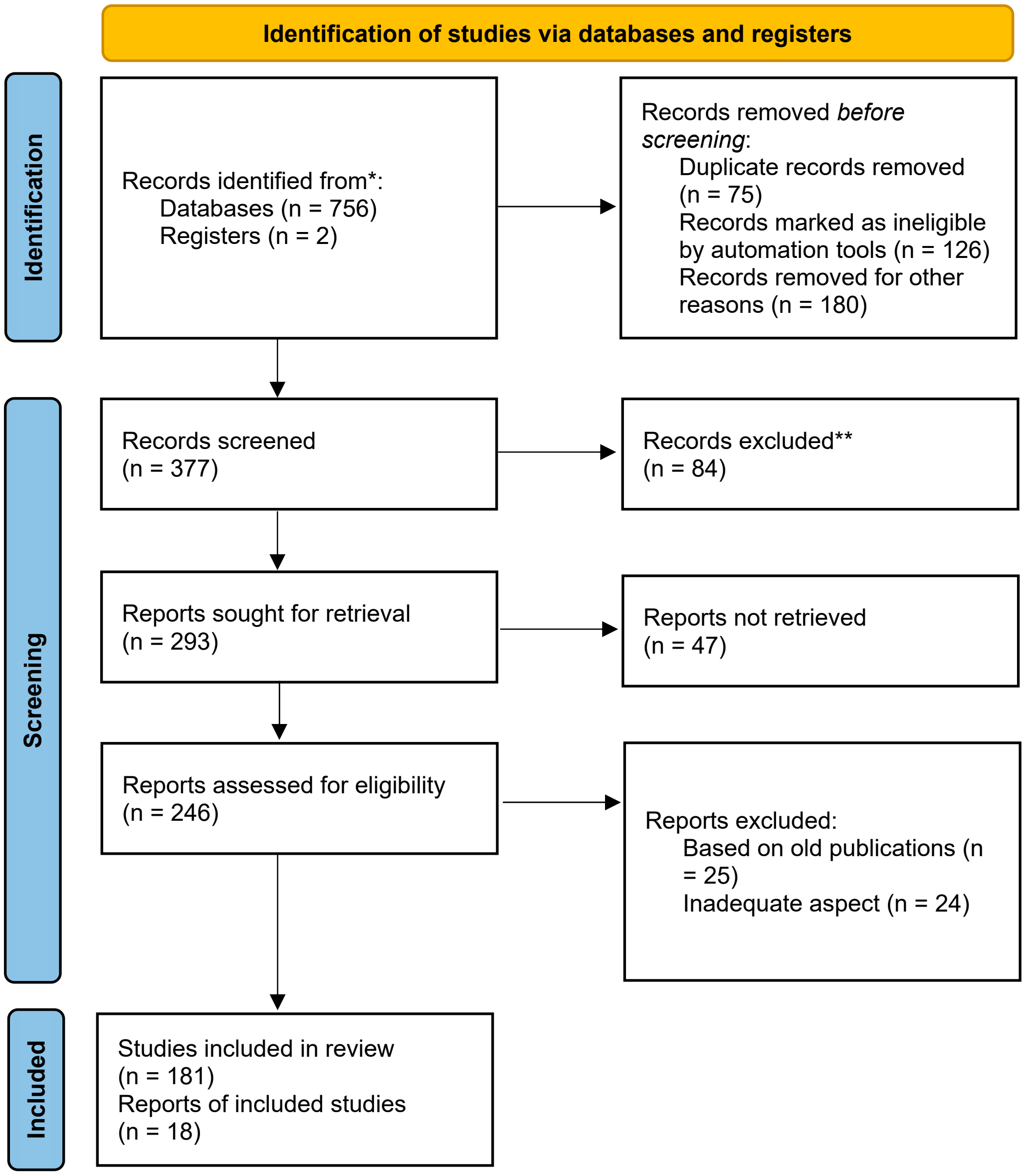

*Consider, if feasible to do so, reporting the number of records identified from each database or register searched (rather than the total number across all databases/registers).

**If automation tools were used, indicate how many records were excluded by a human and how many were excluded by automation tools.

From: Page MJ, McKenzie JE, Bossuyt PM, Boutron I, Hoffmann TC, Mulrow CD, et al. The PRISMA 2020 statement: an updated guideline for reporting systematic reviews. BMJ 2021;372:n71. doi: 10.1136/bmj.n71

For more information, visit: http://www.prisma-statement.org/
